# Metabolic parameters in cord blood of neonate of mothers with obese and non-obese PCOS and controls: retrospective cohort study

**DOI:** 10.1186/s12884-021-03697-6

**Published:** 2021-03-20

**Authors:** Sanaz Alizadeh, Shahideh Jahanian Sadatmahalleh, Fatemeh Razavinia, Mahnaz Bahri Khomami, Malihe Nasiri, Ashraf Moini, Saeideh Ziaei

**Affiliations:** 1 Department of Reproductive Health and Midwifery, Faculty of Medical Sciences, Tarbiat Modares University, P.O. Box: 1415-111, Dr. Saeideh Ziaei, Tehran, Iran; 2 Monash Centre for Health Research and Implementation, School of Public Health and Preventive Medicine, Monash University, Clayton, Vic Australia; 3 Department of Biostatistics, Faculty of Paramedical Sciences, Shahid Beheshti University of Medical Sciences, Tehran, Iran; 4 Breast Disease Research Center (BDRC), School of Medicine, Tehran University of Medical Science, Tehran, Iran; 5 Department of Obstetrics and Gynecology, Tehran University of Medical Sciences, Tehran, Iran; 6 Department of Endocrinology and Female Infertility, Reproductive Biomedicine Research Center, Royan Institute for Reproductive Biomedicine, ACECR, Tehran, Iran

**Keywords:** Polycystic ovary syndrome, Metabolic parameters, Neonate, Pregnancy

## Abstract

**Background:**

Polycystic ovary syndrome (PCOS) is characterized by reproductive disorder and increased risk of metabolic syndrome**.** This study aimed to assess the metabolic parameters in the cord blood of neonate of mothers with obese PCOS and comparison with non-obese PCOS and controls.

**Methods:**

This retrospective cohort study was conducted in Arash and Kamali Hospital in 2017–2018. The biochemical test was conducted on 78 neonates from obese PCOS mothers, 78 neonates from non-obese PCOS mothers, and 78 neonates from healthy mothers. Finally, cord blood lipid profile and insulin and blood sugar were determined by specific kits. Correlations between variables were compared with chi-square, Mann-Whitney’s U, Kruskal-Wallis H tests and regression model by SPSS 23 and *P* < 0.05 was considered significant.

**Results:**

Triglycerides (TG) and high-density lipoprotein cholesterol (HDL) were higher in cord blood of newborn of obese PCOS women than non-obese PCOS and controls (*P* = 0.02, *P* < 0.001, respectively). Also, the mean insulin was higher in cord blood of neonate of non-obese PCOS women than in obese PCOS and controls (12.26 ± 12.79 vs. 11.11 ± 16.51 vs. 6.21 ± 10.66, *P* = 0.01). But in the study, there was no significant difference between the mean of umbilical cord low-density lipoprotein cholesterol (LDL), total cholesterol and blood sugar in three groups. The logistic regression model showed that metabolic parameters were related to PCOS.

**Conclusions:**

In the present study, there was a significant difference between the mean of umbilical cord HDL, cholesterol, and the insulin level in the three groups. But, there was no significant association between the mean of blood sugar, LDL, and TG in the groups. The metabolic disorder in PCOS might affect cord blood lipid and insulin and adulthood health.

## Background

Polycystic ovary syndrome (PCOS), affecting 6–10% reproductive-aged women, is one of the most common endocrine disorders [1]. It is a multifactorial condition related to gens and environmental risk factors [2]. PCOS is associated with hormonal dysregulation such as hyperandrogenemia and disturbed gonadotropin secretion [2]. These result in chronic anovulation, and menstrual disorder obesity and insulin resistance, type II diabetes mellitus, dyslipidemia, hyperlipidemia, and cardiovascular disease [3]. Metabolic syndrome is reported in 7–43% of women with PCOS [4–6]. Metabolic syndrome is associated with insulin resistance, hypertension, obesity, lower HDL, higher LDL, and increased fasting blood sugar (FBS) [5]. Metabolic syndrome and PCOS are both associated with increased rate of adverse neonatal and maternal pregnancy outcomes including gestational diabetes mellitus (GDM), hyperlipidemia, hypertension in mother, and maternal obesity and diabetes have been associated with macrosomia, risk for obesity, glucose intolerance and anderogen level disorder of the infant and the offspring [7].

Recent data report that there is an association between intrauterine environment (such as hyperglycemia, hyperandrogenism and hyperlipidemia) and health status in adulthood [8–10]. Therefore, maternal hormonal imbalance and dyslipidemia may have adverse short and long term impact on infant’s health. Recent studies have shown that maternal PCOS increases testosterone in the infant [11]. Increased testosterone levels in infant cause vascular endothelial damage [11], hypertension and cardiovascular disease in adulthood [12, 13]. Also, Maternal obesity and PCOS lead to increased blood glucose and lipids [14], type 2 diabetes [15], neonatal and adolescent obesity [16], and increased risk of developing PCOS and metabolic syndrome in offspring [17]. increase disease (diabetes, obesity, cardiovascular disease) and economic and psychological burden in the person, in the family and in the society [18].

As a result, due to the importance of the issue, we aimed to assess the metabolic parameters (LDL and HDL, triglycerides, cholesterol, blood sugar, and insulin) in the cord blood of neonate of mothers with obese PCOS and comparison with non-obese PCOS and controls.

## Methods

This retrospective cohort study was conducted among pregnant women in Arash Hospital in Tehran and Kamali Hospital in Karaj from 2017 to 2018. The study protocol was approved by the ethics committee of Tarbiat Modares University (IR.TMU.REC.1395.367). Informed consent was obtained from all participants.

Using the equality of means in three groups formula, sample size is calculated by taking 99% confidence coefficient and power 90% and effect size 0.3 [17]. In this study, 234 women- neonate were enrolled in the study according to the inclusion criteria and available method and divided into three groups (78 neonates from obese PCOS mothers, 78 neonates from non-obese PCOS, and 78 neonates from healthy mothers). Also, informed consent was obtained from all participating mothers.

In this study, we recruited 18–40 years women with a spontaneous singleton pregnancy, Iranian nationality and ability to read and write (*n* = 250). We excluded those with chronic metabolic (such as phenylketonuria, thyroid, diabetes and etc) and non-metabolic diseases (such as cardiovascular disease, chronic kidney disease and etc), a history of chemotherapy or radiotherapy, hyperprolactinemia or androgen-secreting neoplasms, late-onset 21-hydroxylase deficiency, adult-onset congenital adrenal hyperplasia, the Cushing’s syndrome, and thyroid disease and also smokers (*n* = 16).

PCOS is diagnosed in those with having at least two of the following three symptoms before pregnancy: oligomenorrhea or amenorrhea (O), clinical and/or biochemical hyperandrogenism (H), and polycystic ovaries on ultrasound (P) [19].

The controls were healthy women with regular menstrual cycles. They had no history of any long-term medication intake (due to its effects on PCOS symptoms and diseases, lipids and blood glucose, or drug side effects (obesity, liver dysfunction, etc.)), hyperandrogenemia, hirsutism, gestational diabetes, thyroid dysfunction, galactorrhea, and hypertension.

Infants with genetic disorders, abnormalities, or preterm birth were excluded from the study.

In each PCOS group, 31 male and 29 female neonates were examined. As a control group, we examined 34 male and 26 female infants born to healthy mothers. Newborns in both groups (control and PSCO) had normal birth weight.

The three groups (control, obese and non-obese PCOS) were group matched for age, education, employment, social-economic status, parity, and gestational age at labor, as well as the neonate’s sex. Neonates with genetic disorders, malformation or preterm delivery were excluded from the study.

We finally included 234 pregnant women in the analyses. Women with PCOS were then categorized to those with (pre-pregnancy BMI > 30) and without obesity (pre-pregnancy BMI < 30). Pre-pregnancy weight and BMI was reported from the subject’s final doctor’s visit or on admission to the hospital.

### Laboratory measurements

The umbilical cord blood samples (5 ml) were collected immediately post-delivery by researcher. The samples were centrifuged and the serum was separated and frozen at − 70 °C until processed for the metabolic index in the laboratory. Serum levels of triglycerides (TG) and cholesterol were determined by standard colorimetric assays (Pars Azmoon, Iran), LDL and HDL were measured by immunoturbidometric. Also, serum levels of insulin were determined by chemiluminescent immunoassay (CLIA) (DiaSorin, Saluggia, Italy), blood sugar concentration was measured by photometric.

### Statistical analysis

Statistical analysis was performed by using Statistical Package for Social Science (SPSS, version 23, SPSS Inc., Chicago, IL, USA).

The normality assumption of variables was tested by using the Kolmogorov-Smirnov test and data are presented as mean ± SD, number (percentage) and median (IQR1-IQR3). Also, correlations between variables were compared with chi-square, tukey test, One Way ANOVA and Kruskal-Wallis H tests. In addition, to detect the association between metabolic parameters in cord blood of neonate and PCOS with Logistic regression model with Logit link function (to estimate the odds ratio of metabolic parameters with 95% confidence intervals) was run. In this study, *P* < 0.05 was considered significant.

## Results

The demographic characteristics of the obese PCOS, non-obese PCOS and control groups are presented in Table 1. There was no significant difference between demographic characteristics in the three groups (Table 1).

**Table 1:** Homogeneity of three groups of obese and non-obese PCOS mothers and controls in terms of demographic characteristics

Variables	Obese mothers with PCOS (***n*** = 78)	Non-obese mothers with PCOS (***n*** = 78)	Control (***n*** = 78)	***P***-value
**Age (years)**^**a**^	28.4 ± 5.16	27.7 ± 4.54	26.4 ± 5.18	0.089
**BMI (kg/m**^**2**^**)**^**a**^	31.10 ± 3.11	26.06 ± 4.18	25.18 ± 2.93	**0.01**
**Education**^**b**^				
**Diploma (< 12 years)**	64 (82.1)	66 (84.6)	73 (93.6)	0.07
**College education (≥12 years)**	14 (17.9)	12 (15.4)	5 (6.4)	
**Income (Rial)**^**b**^				
**(2,000,000)**	23 (29.5)	20 (25.6)	28 (35.9)	0.46
**(2,000,000–3,000,000)**	33 (42.3)	37 (47.4)	36 (46.2)	
**(> 3,000,000)**	22 (28.2)	21 (27)	14 (17.9)	
**Gravid**^**b**^				
**1**	30 (38.5)	39 (49.9)	30 (38.5)	0.29
**2**	27 (34.6)	25 (32.1)	31 (39.7)	
**3**	14 (17.9)	9 (11.5)	12 (15.4)	
**4**	7 (9)	5 (6.5)	5 (6.4)	
**Parity**^**b**^				
**1**	50 (65)	58 (69.85)	50 (65)	0.625
**2**	20 (25)	15 (18.75)	18 (22.5)	
**3**	8 (10)	9 (11.4)	10 (12.5)	
**Infants Gender**^**c**^				
**Male**				
**Female**				

*PCOS* Polycystic Ovary Syndrome, ^a^Values are given as mean ± SD using One Way ANOVA, ^b^Values are given as a number (%) using Kruskal Wallis test,^c^

According to the Table 1, there was a significant difference between the mean of BMI in the three groups. Tukey test results showed that the two-to-two comparison between the groups was significant (*p* < 0.001) and the mean BMI in the control group was lower than other two groups and in the obes women is higher than other two groups.

As shown in Table 2, the mean rank serum level of insulin in the cord blood of neonates of mothers in three groups was significant (*P* = 0.01). Also, Mann-Whitney U test showed that a statistically significant between insulin in obese PCOS and control group (*P* < 0.001) and non-obese PCOS and control group (*P* = 0.003). (Based on Bonferroni correction, a significance level of 1% was considered).

**Table 2:** Metabolic Parameters in cord blood of obese and non-obese PCOS mothers and controls

Variables	Obese mothers with PCOS (***n*** = 78)	Non-obese mothers with PCOS (***n*** = 78)	Control (***n*** = 78)	***P***-value
Insulin (μIU/ml)^a^	5.40 (1.70–14.40)	9.50 (3.55–18.55)	2.45 (1.60–6.05)	**0.01**
Blood Suger (mg/dl)^a^	74.50 (54.25–91.50)	73.50 (53.00–92.50)	77.00 (53.25–100.00)	0.41
TotalCholosterol (mg/dl)^a^	73.00 (56.25–83.50)	70.50 (58.00–85.75)	65.00 (55.25–79.00)	0.22
HDL Cholesterol (mg/dl)^a^	30.50 (16.25–45.00)	21.00 (18.00–31.75)	30.50 (16.25–45.00)	**< 0.001**
LDL Cholesterol (mg/dl)^a^	21.00 (16.00–28.75)	23.00 (15.00–28.00)	21.00 (18.00–29.00)	0.71
Triglycerides (mg/dl)^a^	45.50 (34.25–65.50)	45.00 (32.25–59.00)	31.00 (19.00–48.50)	**0.02**

*PCOS* Polycystic Ovary Syndrome

^a^Values are given as median (IQR1-IQR3) using Kruskal Wallis

The mean rank umbilical cord TG level in three groups was significant(*p* = 0.02) and Mann-Whitney U test showed that TG in obese PCOS and control (*P* = 0.008) and non-obese PCOS and control (*P* < 0.001) was significant.

The mean rank serum level of HDL in obese PCOS and non-obese PCOS and the control group was statistically significant (*P* < 0.001). And the umbilical cord HDL in control was significantly lower compared with two groups (*P* < 0.001).

In the present study, there was no significant difference between the mean rank of umbilical cord LDL (*P* = 0.71), cholesterol (*P* = 0.22) and sugar (*P* = 0.41) level in the three groups.

Logistic regression model showed metabolic parameters insulin (OR = 1.048, 95% CI: 1.008–1.090), *P* = 0.01), TG (OR = 1.024, 95% CI: 1.009–1.039), *P* = 0.002) in obese PCOS women and insulin (OR = 1.053,95%CI:1.012–1.095), *P* = 0.01), HDL (OR = 0.967, 95% CI: 0.948–0.987), *P* = 0.001) and TG (OR = 1.026, 95% CI: 1.011–1.041), *P* = 0.001) in non-obese PCOS women) were related to PCOS. And one unit increase in insulin level increases 4.8% odds of obese PCOS and 5% non-obese PCOS compared to controls. Also, One unit increase in TG level, the chance of obese PCOS at 2.4% and the chance of non-obese PCOS at 2.6% was higher than control. But, one unit increase in HDL level, decreases 3.3% chance of non-obese PCOS compared to controls (Table 3).

**Table 3:** Predictors of metabolic syndrome in obese PCOS, non-obese PCOS, and controls women using logistic regression model

Variables	β	Odds Ratio	95% CI	***P***-value
**Obese mothers with PCOS**				
Insulin (μIU/ml)	0.048	1.048	1.008–1.090	**0.018**
Blood Suger (mg/dl)	0.003	1.003	0.997–1.009	0.325
Total Cholosterol (mg/dl)	0.016	1.016	0.997–1.036	0.102
HDL Cholesterol (mg/dl)	−0.001	0.999	0.991–1.007	0.822
LDL Cholesterol (mg/dl)	−0.027	0.973	0.936–1.011	0.156
Triglycerides (mg/dl)	0.024	1.024	1.009–1.039	**0.002**
**Non-obese mothers with PCOS**				
Insulin (μIU/ml)	0.053	1.053	1.012–1.095	**0.010**
Blood Suger (mg/dl)	−0.001	0.999	0.991–1.007	0.816
Total Cholosterol (mg/dl)	0.013	1.013	0.933–1.035	0.206
HDL Cholesterol (mg/dl)	−0.033	0.967	0.948–0.987	**0.001**
LDL Cholesterol (mg/dl)	−0.018	0.982	0.945–1.021	0.357
Triglycerides (mg/dl)	0.026	1.026	1.011–1.041	**0.001**

*PCOS* Polycystic Ovary Syndrome, *OR* odds ratios, *CI* confidence intervals

## Discussion

The current study aimed at assessing the umbilical cord blood’s HDL, LDL, TG, cholesterol, insulin, and sugar level in the newborns of PCOS mothers compared to the controls.

In the present study, there was a significant difference between the mean of umbilical cord TG and HDL levels in the women PCOS (obese PCOS and non-obese) and controls. However, there was no association between total cholesterol and LDL levels in the groups. In several studies including Mehrabian et al. [20], observed a significant relationship between serum TG and LDL levels in neonates of PCOS women compared to controls, but, there was no difference between serum cholesterol and HDL levels. Also, Maliqueo et al. [21], reported not the association between serum levels of cholesterol, TG, HDL and LDL in the cord blood of neonates with PCOS and controls. The difference in the findings of these three studies might be because of the ethnic differences in cord blood lipid profile, and anthropometric differences in the neonate, type of delivery, gestational age, race, lifestyle, diet and etc.

At least half of the patients with PCOS are obese. In addition, over 50% of PCOS women have android obesity. Android obesity increases cardiovascular disease risk and decreases HDL. On the other hand, in obese women, sex hormone-binding globulin (SHBG) decreases and testosterone increases. Increasing testosterone decreases lipoprotein lipase activity in abdominal adipose cells and disrupts the anti-lipolytic effect of insulin [22, 23]. And obesity causes insulin resistance and hyperinsulinemia. Insulin resistance and hyperinsulinemia play an important role in dislipidemia (the degree and type of dyslipidemia vary in women with PCOS). Hyperinsulinemia inhibits lipolysis and increases non-esterified fatty acid. Increasing non-esterified fatty acid increases TG and decreases HDL [2, 23]. Also, increased LDL is of heterogeneous origin in these persons [24].

In this study, serum insulin levels in neonates of non-obese PCOS mothers were higher than obese PCOS and controls. Nevertheless, there was no significant difference between the cord blood sugar levels in the three groups. But, Mehrabian et al. [20], reported no association between umbilical cord insulin levels in the PCOS and controls. Maliqueo et al. [21], observed not the correlation between umbilical cord insulin and glucose levels in the groups. Also, Sergio et al. [25], showed that Serum glucose and insulin levels and insulin resistance were not different in male neonates (2–3 months) with PCOS and non-PCOS women.

The difference in the findings of studies might be because of differences in sample size, genetic variation, nutritional status, cultural differences and lifestyle in the study population. Half of the PCOS people are not obese [12]. But, there is insulin resistance and hyperinsulinemia in all women with PCOS that is not known etiology sofar [26–28]. A sedentary lifestyle [29] and a high-calorie diet [30] may play a role in increasing insulin resistance and blood insulin level (independent of obesity) in PCOS women.

Our study limitations, infant anthropometric was not measured and was not investigated the association with metabolic parameters. Also, the relationship between maternal and neonatal metabolic parameters was not evaluated. In addition, the control group was not divided into obese and non-obese groups. Also, the sample size is small.

## Conclusions

In conclusion, our results indicate a significant association between insulin, HDL, TG in cord blood of neonate of obese PCOS women than in non-obese PCOS and controls. But, there was no significant difference between the mean of umbilical cord LDL, total cholesterol and blood sugar in three groups. The metabolic disorder in PCOS might affect cord blood lipid and insulin. These disorders may be an increased risk for chronic diseases (cardiovascular disease, Diabet, etc) in adulthood. As a result, screening in PCOS women prevents neonatal and adult complications.

## Data Availability

All data generated and analyzed in this study are included in this published manuscript.

## Abbreviations

FBS: Fasting blood sugar
GDM: Gestational diabetes mellitus
HDL: High-density lipoprotein cholesterol
LDL: Low-density lipoprotein cholesterol
PCOS: Polycystic ovary syndrome
SHBG: Sex hormone-binding globulin
TG: Triglycerides
